# Causal relationship between PCSK9 inhibitor and common neurodegenerative diseases: A drug target Mendelian randomization study

**DOI:** 10.1002/brb3.3543

**Published:** 2024-06-05

**Authors:** Qiang Huang, Qin Zhang, Bei Cao

**Affiliations:** ^1^ Department of Neurology West China Hospital Sichuan University Chengdu Sichuan China; ^2^ Department of Neurology The First People's Hospital of Jinzhong Jinzhong Shanxi Province China

**Keywords:** drug target Mendelian randomization, HMGCR, neurodegenerative diseases, PCSK9

## Abstract

**Background:**

In addition to lowering cholesterol levels, the proprotein convertase subtilis kexin 9 (PCSK9) inhibitor has a variety of effects, including anti‐neuroapoptosis. However, the effects of PCSK9 inhibitors on neurodegenerative diseases are controversial. Therefore, we used drug‐targeted Mendelian randomization (MR) analysis to investigate the effects of PCSK9 inhibitors on different neurodegenerative diseases.

**Methods:**

We collected single nucleotide polymorphisms (SNPs) of PCSK9 from published statistics of genome‐wide association studies and performed drug target MR analyses to detect a causal relationship between PCSK9 inhibitors and the risk of neurodegenerative diseases. We utilized the effects of 3‐Hydroxy ‐3‐ methylglutaryl‐assisted enzyme A reductase (HMGCR) inhibitors (statin targets) for comparison with PCSK9 inhibitors. Coronary heart disease risk was used as a positive control, and primary outcomes included amyotrophic lateral sclerosis (ALS), Parkinson's disease (PD), and Alzheimer's disease (AD).

**Results:**

PCSK9 inhibitors marginally reduced the risk of ALS (OR [95%] = 0.89 [0.77 to 1.00], *p* = 0.048), while they increased the risk of PD (OR [95%] = 1.417 [1.178 to 1.657], *p* = 0.004). However, HMGCR inhibitors increased the risk of PD (OR [95%] = 1.907 [1.502 to 2.312], *p* = 0.001).

**Conclusion:**

PCSK9 inhibitors significantly reduce the risk of ALS but increase the risk of PD. HMGCR inhibitors may be the risk factor for PD.

## Graphical abstract

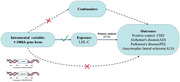

## 1 INTRODUCTION

Neurodegenerative diseases (NDDs) are heterogeneous neurological disorders that can lead to progressive neuronal damage in the central nervous system (CNS) or the peripheral nervous system (PNS), adversely affecting the productive lives of millions of people worldwide. Common neurodegenerative diseases include ALS, PD, AD, and other similar conditions. Currently, the treatment of these diseases mainly focuses on symptomatic therapy, making it difficult to reverse the onset and progression of the diseases. This approach often results in poor therapeutic effects and prognosis, imposing a heavy burden on families and society. Some studies have shown that abnormal lipid metabolism is closely related to neurodegenerative diseases (Hurh et al., 2022; Janse van Mantgem et al., 2023). Therefore, dyslipidemia may be associated with the development and progression of neurodegenerative diseases. There were some evidence suggested that lipid‐lowering drugs have an impact on neurodegenerative diseases (Rajabian et al., 2023). For example, Statin therapy was reported to associate with decreased development of AD (Lee et al., 2020). However, the causal effects of various lipid‐lowering drugs on these conditions require further investigation.

Proprotein convertase subtilisin kexin 9 (PCSK9) is a serine protease that plays an important role in mediating low‐density lipoprotein cholesterol (LDL‐C) metabolism and has emerged as a key target for cholesterol‐lowering therapy (Rosoff et al., 2022). Despite the widely recognized protective effects of PCSK9 inhibitors against cardiovascular disease (CVD), there have been relatively few studies investigating their effects on neurodegenerative diseases. One study demonstrated elevated PCSK9 concentrations in the cerebrospinal fluid and brain autopsies of AD patients compared to controls (Picard et al., 2019), with similar observations reported in other neurodegenerative diseases (Courtemanche et al., 2018). It is noteworthy that consensus is lacking regarding the relationship between genetic mutations in PCSK9 and AD, as PCSK9 loss‐of‐function mutation carriers exhibit a neutral risk of developing AD (Benn et al., 2017; Mefford et al., 2018; Paquette et al., 2018). Furthermore, the impact of PCSK9 on other neurodegenerative diseases (ALS, PD) has not been thoroughly investigated, despite the acknowledged association between lipid metabolism and the severity of these diseases (Hurh et al., 2022; Janse van Mantgem et al., 2023). Notably, PCSK9 inhibitors exhibit potential pleiotropic effects beyond lipid‐lowering, distinguishing them from conventional lipid‐lowering drugs such as HMGCR inhibitors (HMGCRi). PCSK9 interferes with the anti‐apoptotic signaling pathway by modulating the Apolipoprotein E Receptor 2 (ApoER2), thereby promoting neuronal apoptosis (Bingham et al., 2006; Kysenius et al., 2012). These findings imply that PCSK9 inhibitors (PCSK9i) may play a role in the pathogenesis of neurodegenerative diseases through mechanisms beyond lipid lowering. However, the role of PCSK9 inhibitors in different neurodegenerative diseases may be inconsistent and requires further exploration.

Mendelian randomization (MR) analyses of drug targets utilize genetic variations that mimic the pharmacological inhibition of a drug's genetic target as instrumental variables. Regression analyses have been instrumental in elucidating the effects of these drugs, which have been in use for extended periods, and in illustrating the potential impact of drug action targets on neurodegenerative diseases (Burgess et al., 2017). This study collected summary statistics from recently published genome‐wide association studies (GWAS) via a pharmacological target Mendelian randomization study to investigate the causal relationship between PCSK9 inhibitors, HMGCR inhibitors, and neurodegenerative diseases, including amyotrophic lateral sclerosis (ALS), Parkinson's disease (PD), and Alzheimer's disease (AD).

## 2 METHODS

### 2.1 Instrumental variables for screening PCSK9 and HMGCR

**FIGURE 1:**
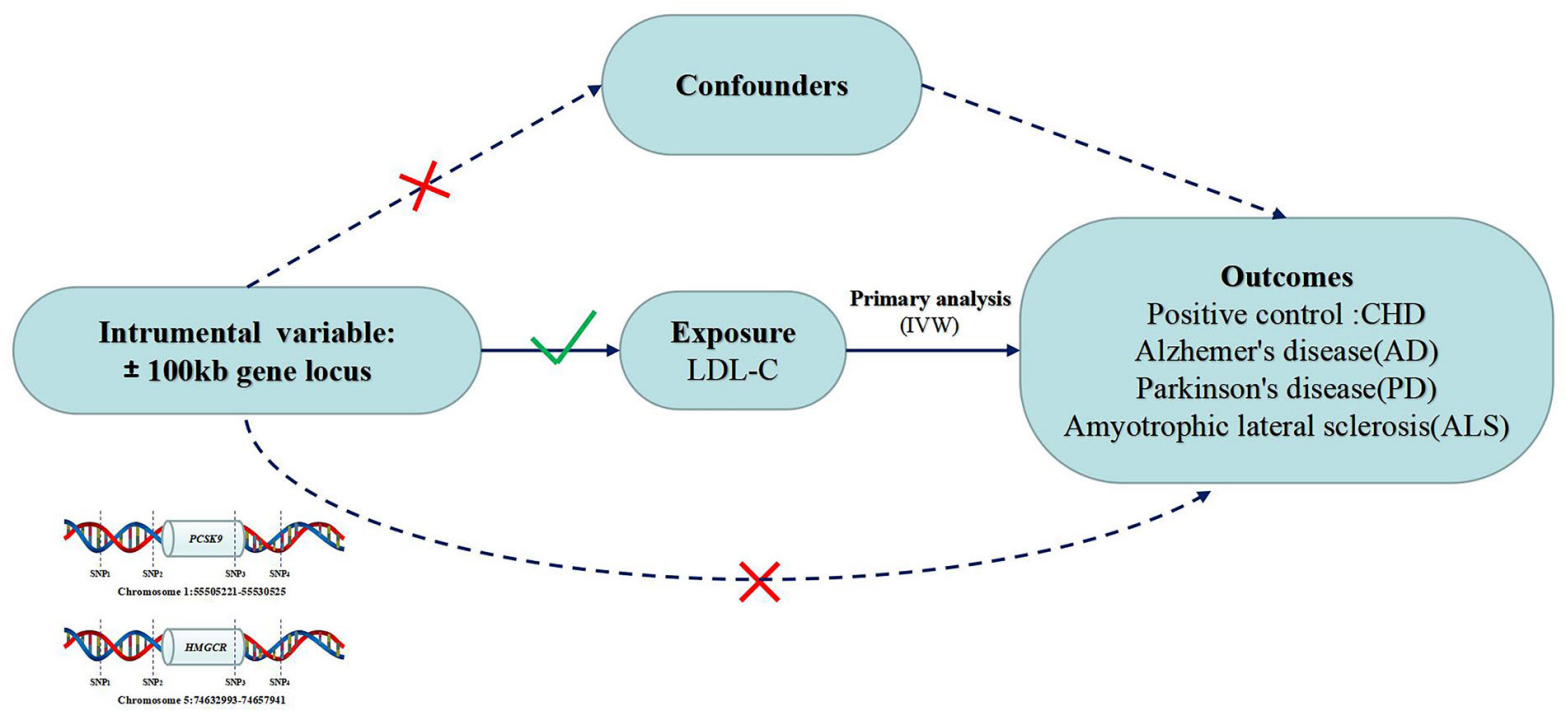
Design flow chart of the present study.

Pooled data on LDL‐C were obtained from a GWAS summary statistic containing 440,546 Europeans (Richardson et al., 2020). PCSK9 inhibitors and HMGCR inhibitors (statins) were used as instrumental variables to model the effects of PCSK9 and HMGCR on lowering LDL‐C (Richardson et al., 2020). Single nucleotide polymorphisms (SNPs) located within ± 100 kb of the PCSK9 or HMGCR locus and associated with LDL‐C levels were selected as instrumental variables (**Figure** **1**). To mitigate the impact of strong linkage disequilibrium (LD) on the outcomes, a threshold of (*r* ^2^ < 0.3) was set. Ultimately, 13 SNPs significantly associated with PCSK9 and 8 SNPs significantly associated with HMGCR were retained (**see** **Table** **S1**).

### 2.2 The source of the outcomes

Four diseases were used as endpoints for the drug‐targeting analysis, with CHD serving as a positive control. All data were obtained from European populations. Data for CHD were obtained from GWAS summary statistics comprising 42,096 cases and 99,121 controls (Nikpay et al., 2015). Additionally, we collected ALS (van Rheenen et al., 2021), PD (Sakaue et al., 2021), and AD GWAS data (https://gwas.mrcieu.ac.uk/) for analysis.

### 2.3 Data analysis

PCSK9 inhibitors and HMGCR inhibitors are widely utilized in the treatment of CHD. Hence, we utilized GWAS data of CHD as a positive control to validate the instrumental variables. Initially, we integrated the exposure‐related drug‐targeting instrumental variables with the outcome dataset. Subsequently, we conducted analyses using MR Egger, weighted median, inverse variance weighted (IVW), simple mode, weighted mode, and MR‐PRESSO, with IVW being the primary analysis method (Wang et al., 2022). MR Egger was employed to test for heterogeneity in the data, with Cochrane's Q value used to assess heterogeneity of the instrumental variables. A *p*‐value > .05 indicated no significant heterogeneity. MR Egger regression equation was utilized to assess the presence of horizontal polytropy in genetic instruments, with a *p*‐value > .05 indicating no horizontal polytropy (Hemani et al., 2018). SNPs in MR must satisfy the assumption of not being directly correlated with the outcome (**see** **Figure** **1**). Consequently, we utilized the online platform LDTrait (https://ldlink.nih.gov/?tab = ldtrait) to identify traits directly associated with SNPs of the instrumental variables. SNPs associated with ALS, PD, and AD were then excluded. Sensitivity analysis was conducted following outlier removal via the MR‐PRESSO test. To ensure robustness against individual SNP influence, we employed the leave‐one‐out method to iteratively remove each SNP and compared the IVW method results with all variants. Also, Steiger analysis was conducted to detect the reverse causality. Data analysis was conducted using R version 4.3.1 with the MR‐PRESSO and TwoSampleMR packages (Hemani et al., 2018; Verbanck et al., 2018). Results obtained from the IVW method were compared against all variants.

In our study, NDDs outcomes included ALS, PD, and AD. Therefore, a Bonferroni correction was utilized and significant threshold was set as 0.05/6 = 0.008. An IVW *p* < 0.008 was regarded as significant. An IVW *p* >0.008 but <0.05 was regarded as marginal significant.

## 3 RESULTS

### 3.1 Positive control analysis

**FIGURE 2:**
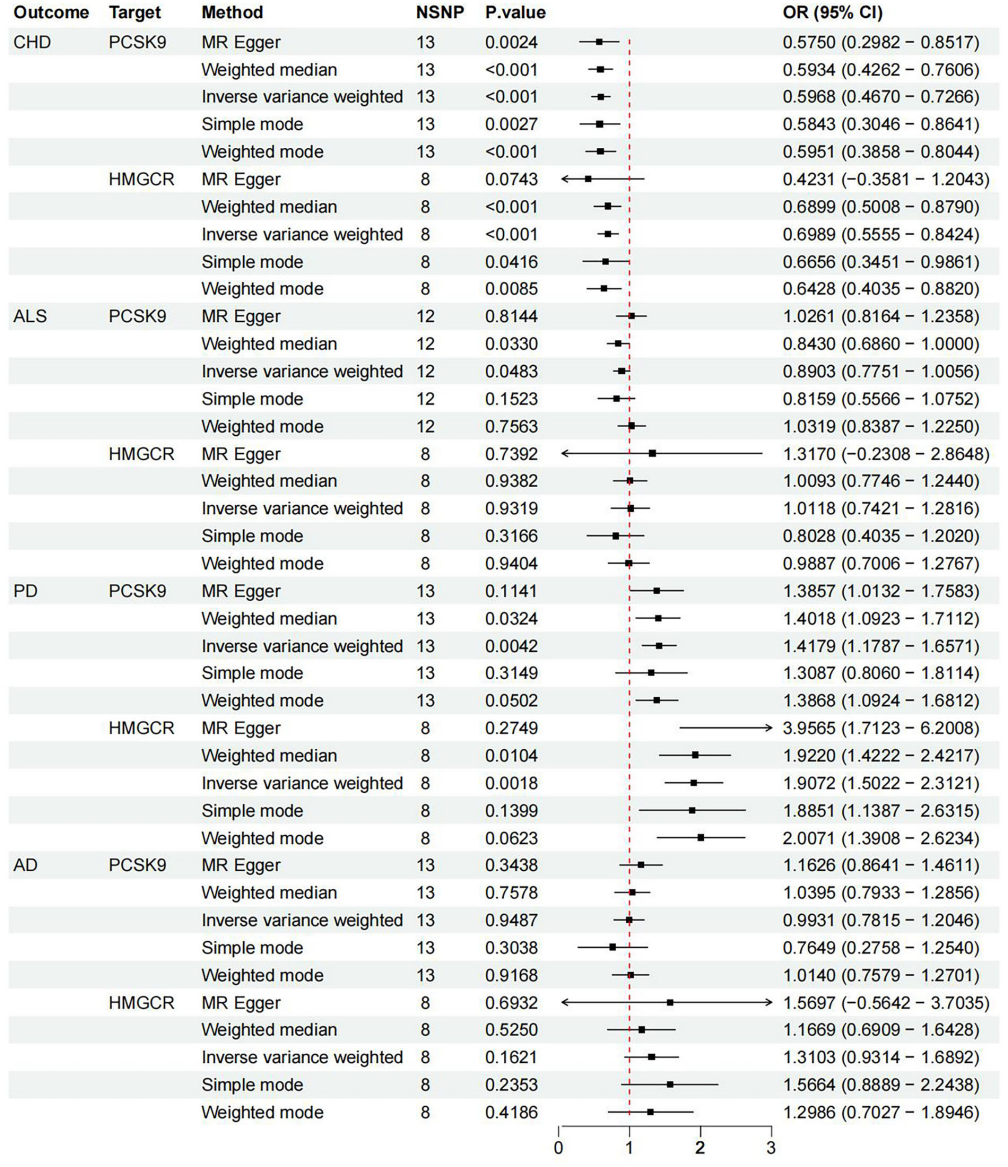
The effect of PCSK9 and HMGCR inhibitor on coronary heart disease and Neurodegenerative diseases.

As anticipated, the application of the IVW approach revealed that PCSK9 inhibitors significantly reduced the risk of CHD (OR [95%] = 0.59 [0.46 to 0.72], *p* = 6.58 × 10^−15^), with a comparable effect observed for HMGCR inhibitors (OR [95%] = 0.69 [0.55 to 0.84], *p* = 9.85 × 10^−7^) (see **Figure** **2**). Results of the MR Egger, weighted median, simple mode, weighted mode, and MR‐PRESSO analyses are provided in **Table** **S2**.

### 3.2 The causal relationship between PCSK inhibitors, HMGCR inhibitors and neurodegenerative diseases

PCSK9 inhibitors demonstrated a marginal significant protective effect against ALS in both IVW (OR [95%] = 0.89 [0.77 to 1.00], *p* = 0.048) and weighted median (OR [95%] = 0.84 [0.68 to 0.99], *p* = 0.032) analysis methods. The direction of MR Egger and weighted mode was inconsistent with the IVW, WM, and simple mode. Therefore, the significant conclusion should be cautious. In contrast, HMGCR inhibitors did not achieve statistical significance (IVW: *p* = 0.93; weighted median: *p* = 0.93). Results from other MR analysis methods will be presented in **Table** **S2**. Furthermore, both PCSK9 inhibitors and HMGCR inhibitors were associated with an increased risk of developing PD (PCSK9: IVW: OR [95%] = 1.417 [1.178 to 1.657], *p* = 0.004; HMGCR: IVW: OR [95%] = 1.907 [1.502 to 2.312], *p* = 0.001). Nonetheless, neither PCSK9 inhibitors nor HMGCR inhibitors were significantly associated with the risk of AD (**see** **Table** **S2** **and** **Figure** **2**).

### 3.3 Sensitivity analysis

**FIGURE 3:**
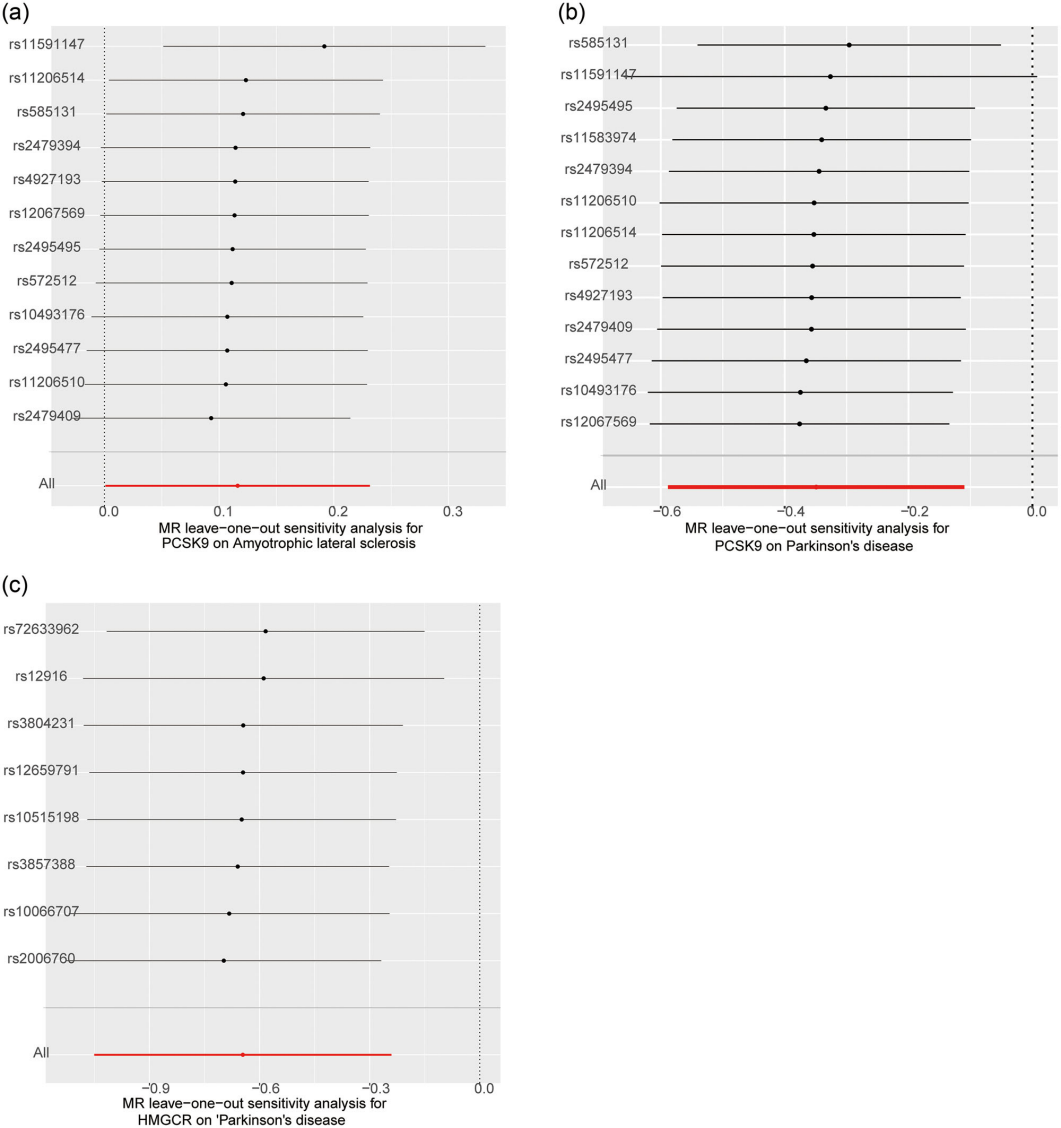
Leave‐one‐out sensitivity analysis for PCSK9 on Amyotrophic lateral sclerosis (a) and Parkinson's disease (b) and HMGCR on Parkinson's disease (c).

Cochrane's Q and MR Egger regression equation were utilized to assess the level of heterogeneity and horizontal pleiotropy (**see** **Table** **S3**). Additionally, the sensitivity analysis results indicated no heterogeneity or horizontal pleiotropy in any of the outcomes (*p* > 0.05) (**see** **Table** **S3**). The leave‐one‐out method were presented in **Figure** **3**. No SNPs were found to related to ALS, PD, and AD. Steiger analysis indicated that our analysis was not biased by reverse causality.

## 4 DISCUSSION

The PCSK9 inhibitor has been widely used as a novel LDL‐C lowering drug for the prevention and treatment of CVD in the clinic (Gallego‐Colon et al., 2020; Hackam & Hegele, 2022). Besides its central role in LDL‐C regulation and CVD, PCSK9 has been implicated in various biological processes such as infectious shock, vascular inflammation, viral infections, and cancer (Seidah & Garcon, 2022). PCSK9 has garnered increasing attention for its involvement in neurodegenerative diseases (Benn et al., 2017; Hurh et al., 2022; Paquette et al., 2018). To date, there has been a lack of comprehensive and systematic studies addressing the causal relationship between PCSK9 and the risk of developing neurodegenerative diseases. Our drug‐targeted MR analysis revealed that the PCSK9 inhibitor significantly decreased the risk of ALS, while potentially increasing the risk of PD. These findings contribute significantly to the existing body of research and provide valuable insights for lipid‐lowering drug selection strategies.

Our study demonstrates a significant protective effect of PCSK9 inhibitors against amyotrophic lateral sclerosis (ALS). ALS is a rapidly progressive neurodegenerative disease characterized by the loss of upper motor neurons in the motor cortex, lower motor neurons in the brainstem, and spinal cord, ultimately leading to respiratory failure approximately 3 to 5 years after symptom onset (Tzeplaeff et al., 2023). While the effects of PCSK9 in ALS have not been extensively studied, there is a recognized association between lipid metabolism and ALS severity (Janse van Mantgem et al., 2023). However, the precise mechanisms underlying PCSK9 involvement in ALS onset and progression remain unknown. Our findings suggest that PCSK9 inhibitors reduce the risk of developing ALS through pathways beyond lipid lowering, as evidenced by the lack of a similar effect with HMGCR inhibitors. Since PCSK9 was initially identified in apoptotic primary cerebellar neurons(Chiang et al., 2001; Seidah et al., 2003), it exhibits a property that promotes neuronal apoptosis(Bingham et al., 2006; Kysenius et al., 2012); this may be a potential mechanism of action for PCSK9 inhibitors to protect against ALS. However, PCSK9 exhibited anti‐apoptotic properties in U251 human glioma cells. PCSK9 gene silencing resulted in cell shrinkage, nuclear breakage, and loss of membrane integrity, while PCSK9 overexpression in these glioma cells restored normal morphology (Piao et al., 2015). Thus, previous studies have shown conflicting roles of PCSK9 in apoptosis, with our study highlighting its potential neuroprotective role against ALS. Interestingly, our study revealed an increased risk of Parkinson's disease (PD) associated with PCSK9 inhibitors, consistent with the negative association between serum lipoprotein levels and PD risk over time (Hurh et al., 2022). Additionally, HMGCR inhibitors were also found to increase PD risk, implying a potential lipid‐related mechanism. AD is one of the most common neurodegenerative disorders. β‐amyloid (Aβ) is a major component of neuritic plaques in Alzheimer's disease (AD), and its accumulation has been suggested to be a molecular driver for the pathogenesis and progression of Alzheimer's disease. Aβ has been a major target for Alzheimer's disease treatment (Zhang et al., 2023). A growing number of studies have shown a close relationship between PCSK9 and AD (Picard et al., 2019; Seidah et al., 2003). For example, it has been shown that PCSK9 overexpression decreased BACE1 expression, whereas PCSK9 inhibition increased levels of BACE1 and Aβ deposition (Jonas et al., 2008). Studies in a rat stroke model found that PCSK9 expression was negatively correlated with amyloid plaques (Apaijai et al., 2019). However, another study failed to determine a direct effect of PCSK9 on BACE1 expression or Aβ levels in mice (Liu et al., 2010). Notably, there is no consensus on the association between PCSK9 gene variants and AD, as PCSK9 loss‐of‐function mutation carriers have a neutral risk of developing AD (Benn et al., 2017; Mefford et al., 2018; Paquette et al., 2018). Similarly, our study did not find a causal relationship between PCSK9 suppression and AD risk, nor did it observe such a relationship with HMGCR inhibitors. This suggests that PCSK9's influence on AD risk may not be mediated solely by lipid levels. This is contrary to the major existing studies. Some key points should be noted: one being that previous studies were based on traditional observational designs that are susceptible to confounding factors or reverse causation, and thus the observed associations may be biased. Another reason is that the current study uses limited GWAS summary statistics, which do not allow for more external data validation, which may affect the MR results. However, given the conflicting results, further research is warranted to elucidate the underlying mechanisms and provide more conclusive evidence.

To the best of our knowledge, this is the first relatively systematic MR study investigating the causal relationship between PCSK9 inhibitors and the risk of neurodegenerative diseases. It is important to acknowledge that our study has several unavoidable limitations. First, it is important to note that MR studies cannot replace clinical trials in the real world, as they solely serve as a method to analyze the causal relationship between exposure and outcome. Additional studies are necessary to demonstrate the causal association between PCSK9 and neurodegenerative diseases. Moreover, due to limited GWAS data resources, we conducted MR analyses solely in a European population, precluding external data validation. The efficacy and side effects of PCSK9 inhibitors may vary across different populations. Last, the exposures in our study were expression level of PCSK9 and HMGCR. Therefore, all drugs affect the expression level of these two genes were thought to have effects on NDDs. Exactly effects could be evaluated in the future randomization clinical traits. Hence, future studies should conduct subgroup analyses in diverse populations to draw more comprehensive conclusions.

## 5 CONCLUSIONS

Our drug target MR analysis revealed that PCSK9 inhibitors significantly decreased the risk of ALS but increased the risk of PD. Conversely, HMGCR inhibitors may pose a risk for PD.

## AUTHOR CONTRIBUTIONS

**Qiang Huang**: Formal analysis; software; data curation; project administration; writing—original draft; writing—review and editing. **Qin Zhang**: Supervision; resources; formal analysis; investigation; writing—review and editing. **Bei Cao**: Funding acquisition; conceptualization; methodology; writing—review and editing; visualization; validation.

## CONFLICT OF INTEREST STATEMENT

We declared no conflict of interest.

### PEER REVIEW

The peer review history for this article is available at https://publons.com/publon/10.1002/brb3.3543.

## Supporting information


**Tables S1** The detail of instrumental variable corresponding to PCSK9 and HMGCR.
**Table S2** The effect of PCSK9 and HMGCR inhibitor on neurodegenerative disease.
**Table S3** The result of heterogeneity test and horizontal pleiotropic test.

## Data Availability

All data was publicly available and has mentioned in Section 2.
