# Supplementary material for: Causal relationship between PCSK9 inhibitor and common neurodegenerative diseases: A drug target Mendelian randomization study
Source: Brain Behav. 2024 Jun 5;14(6):e3543. doi: 10.1002/brb3.3543 (PMC11151217; doi:10.1002/brb3.3543)
Supplement: Supplementary file 1 — Tables S1 The detail of instrumental variable corresponding to PCSK9 and HMGCR. Table S2 The effect of PCSK9 and HMGCR inhibitor on neurodegenerative disease. Table S3 The result of heterogeneity test and horizontal pleiotropic test. [file BRB3-14-e3543-s001.docx]

**Supplementary Tables S1** The detail of instrumental variable corresponding to PCSK9 and HMGCR.

|  | SNP | chr | pos | Beta | SE | *p*-value | Effect alleles | Other alleles |
| --- | --- | --- | --- | --- | --- | --- | --- | --- |
| PCSK9 | rs2495495 | 1 | 55496556 | -0.0342 | 0.0059 | 3.52E-08 | C | T |
| PCSK9 | rs2495477 | 1 | 55518467 | -0.064 | 0.0054 | 7.28E-30 | G | A |
| PCSK9 | rs2479409 | 1 | 55504650 | -0.0642 | 0.0041 | 2.51E-50 | A | G |
| PCSK9 | rs12067569 | 1 | 55528629 | 0.0885 | 0.01 | 1.97E-17 | A | G |
| PCSK9 | rs10493176 | 1 | 55538552 | -0.0776 | 0.0102 | 2.54E-14 | G | T |
| PCSK9 | rs11591147 | 1 | 55505647 | -0.497 | 0.018 | 8.57E-143 | T | G |
| PCSK9 | rs4927193 | 1 | 55509872 | -0.0352 | 0.0056 | 4.27E-11 | C | T |
| PCSK9 | rs11583974 | 1 | 55551718 | 0.0646 | 0.0117 | 3.95E-09 | A | G |
| PCSK9 | rs2479394 | 1 | 55486064 | -0.0386 | 0.0041 | 1.58E-19 | A | G |
| PCSK9 | rs11206510 | 1 | 55496039 | -0.0831 | 0.005 | 2.38E-53 | C | T |
| PCSK9 | rs572512 | 1 | 55517344 | 0.0478 | 0.0047 | 5.31E-26 | T | C |
| PCSK9 | rs11206514 | 1 | 55516004 | 0.0507 | 0.0041 | 9.95E-33 | A | C |
| PCSK9 | rs585131 | 1 | 55524116 | 0.0637 | 0.005 | 2.70E-35 | T | C |
| HMGCR | rs3857388 | 5 | 74620377 | 0.0421 | 0.0059 | 2.20E-11 | C | T |
| HMGCR | rs10515198 | 5 | 74641560 | 0.0599 | 0.0061 | 5.99E-22 | A | G |
| HMGCR | rs12659791 | 5 | 74757758 | 0.0433 | 0.005 | 1.42E-18 | C | T |
| HMGCR | rs72633962 | 5 | 74569028 | 0.06 | 0.0072 | 3.33E-15 | C | T |
| HMGCR | rs3804231 | 5 | 74696779 | 0.0642 | 0.0053 | 1.88E-29 | A | G |
| HMGCR | rs10066707 | 5 | 74560579 | 0.0497 | 0.0054 | 2.97E-19 | A | G |
| HMGCR | rs2006760 | 5 | 74562029 | 0.0533 | 0.0076 | 1.67E-13 | G | C |
| HMGCR | rs12916 | 5 | 74656539 | 0.0733 | 0.0038 | 7.79E-78 | C | T |

PCSK9, proprotein convertase subtilisin/kexin 9; HMGCR, 3-hydroxy-3-methylglutaryl coenzyme A reductase

**Supplementary Table S2** The effect of PCSK9 and HMGCR inhibitor on neurodegenerative disease.

| Outcome | Target | Method | NSNP | P.value | OR | OR_LCI95 | OR_UCI95 |
| --- | --- | --- | --- | --- | --- | --- | --- |
| CHD | PCSK9 | MR Egger | 13 | 0.002393164 | 0.574965211 | 0.298236735 | 0.851693688 |
|  |  | Weighted median | 13 | 9.54E-10 | 0.593449035 | 0.426249953 | 0.760648118 |
|  |  | Inverse variance weighted | 13 | 6.58E-15 | 0.59682466 | 0.467002104 | 0.726647217 |
|  |  | Simple mode | 13 | 0.002702216 | 0.584344472 | 0.304562639 | 0.864126305 |
|  |  | Weighted mode | 13 | 0.000391988 | 0.59510778 | 0.385765622 | 0.804449937 |
|  | HMGCR | MR Egger | 8 | 0.07426174 | 0.4230897 | -0.358089193 | 1.204268592 |
|  |  | Weighted median | 8 | 0.000119088 | 0.689895086 | 0.500814074 | 0.878976099 |
|  |  | Inverse variance weighted | 8 | 9.85E-07 | 0.698929046 | 0.555486842 | 0.842371251 |
|  |  | Simple mode | 8 | 0.041635631 | 0.665593546 | 0.345081654 | 0.986105439 |
|  |  | Weighted mode | 8 | 0.008505573 | 0.64275602 | 0.403473495 | 0.882038546 |
| ALS | PCSK9 | MR Egger | 12 | 0.814360193 | 1.026129404 | 0.816427745 | 1.235831064 |
|  |  | Weighted median | 12 | 0.032960422 | 0.843004514 | 0.686041136 | 0.999967891 |
|  |  | Inverse variance weighted | 12 | 0.04826897 | 0.890347806 | 0.775086383 | 1.005609228 |
|  |  | Simple mode | 12 | 0.152300477 | 0.815881446 | 0.556563314 | 1.075199578 |
|  |  | Weighted mode | 12 | 0.75627841 | 1.031853722 | 0.838717931 | 1.224989514 |
|  | HMGCR | MR Egger | 8 | 0.739247972 | 1.316981388 | -0.230828827 | 2.864791602 |
|  |  | Weighted median | 8 | 0.938179521 | 1.009331192 | 0.774612717 | 1.244049668 |
|  |  | Inverse variance weighted | 8 | 0.931854769 | 1.011837174 | 0.742111774 | 1.281562573 |
|  |  | Simple mode | 8 | 0.316632577 | 0.802790783 | 0.403532829 | 1.202048737 |
|  |  | Weighted mode | 8 | 0.940444569 | 0.988684605 | 0.700640753 | 1.276728457 |
| PD | PCSK9 | MR Egger | 13 | 0.11411093 | 1.385712624 | 1.013162833 | 1.758262416 |
|  |  | Weighted median | 13 | 0.032423491 | 1.401766261 | 1.092317802 | 1.711214721 |
|  |  | Inverse variance weighted | 13 | 0.00422616 | 1.417866628 | 1.178650947 | 1.657082309 |
|  |  | Simple mode | 13 | 0.314855458 | 1.308724964 | 0.806003498 | 1.81144643 |
|  |  | Weighted mode | 13 | 0.050179077 | 1.386796046 | 1.092369535 | 1.681222557 |
|  | HMGCR | MR Egger | 8 | 0.274938947 | 3.956538363 | 1.712324261 | 6.200752465 |
|  |  | Weighted median | 8 | 0.01040023 | 1.921962389 | 1.422179224 | 2.421745553 |
|  |  | Inverse variance weighted | 8 | 0.001779302 | 1.907166662 | 1.502207788 | 2.312125536 |
|  |  | Simple mode | 8 | 0.139883445 | 1.885090702 | 1.138730378 | 2.631451026 |
|  |  | Weighted mode | 8 | 0.062272005 | 2.007073249 | 1.390792341 | 2.623354156 |
| AD | PCSK9 | MR Egger | 13 | 0.343832852 | 1.162604672 | 0.864076579 | 1.461132766 |
|  |  | Weighted median | 13 | 0.757789649 | 1.039487621 | 0.793344636 | 1.285630605 |
|  |  | Inverse variance weighted | 13 | 0.948658263 | 0.993073948 | 0.781520737 | 1.204627159 |
|  |  | Simple mode | 13 | 0.303845081 | 0.764856971 | 0.275753684 | 1.253960258 |
|  |  | Weighted mode | 13 | 0.916840594 | 1.014031716 | 0.757914849 | 1.270148583 |
|  | HMGCR | MR Egger | 8 | 0.69316687 | 1.569684591 | -0.564153924 | 3.703523105 |
|  |  | Weighted median | 8 | 0.52504682 | 1.166889038 | 0.690935725 | 1.642842352 |
|  |  | Inverse variance weighted | 8 | 0.162058744 | 1.31032029 | 0.931447761 | 1.689192819 |
|  |  | Simple mode | 8 | 0.235299285 | 1.566366018 | 0.888923146 | 2.243808889 |
|  |  | Weighted mode | 8 | 0.418574623 | 1.298635558 | 0.702667521 | 1.894603595 |

NSNP, number of single nucleotide polymorphisms; OR, odds ratio; CI, confidence interval; PCSK9, proprotein convertase subtilisin/kexin 9; HMGCR, 3-hydroxy-3-methylglutaryl coenzyme A reductase; ALS,amyotrophic lateral sclerosis; PD, Parkinson's disease; AD, Alzheimer disease.

**Supplementary Table S3** The result of heterogeneity test and horizontal pleiotropic test.

| Outcomes | Drug Target | Heterogeneity test | | | | Horizontal pleiotropic test | | |
| --- | --- | --- | --- | --- | --- | --- | --- | --- |
| CHD | PCSK9 | Method | Q | Q_df | Q_pval | egger_intercept | SE | *p*-value |
|  |  | MR Egger | 16.89899 | 11 | 0.1108992 | -0.002818704 | 0.009309443 | 0.767707135 |
|  |  | Inverse variance weighted | 17.03983 | 12 | 0.1481053 |  |  |  |
|  | HMGCR | MR Egger | 3.176024 | 6 | 0.7864519 | -0.029505446 | 0.02302901 | 0.247400659 |
|  |  | Inverse variance weighted | 4.817573 | 7 | 0.6822144 |  |  |  |
| ALS | PCSK9 | MR Egger | 2.749162 | 10 | 0.9867265 | 0.011985296 | 0.007547319 | 0.143365559 |
|  |  | Inverse variance weighted | 5.270970 | 11 | 0.9173366 |  |  |  |
|  | HMGCR | MR Egger | 18.19692 | 6 | 0.0057584 | 0.01553073 | 0.045715928 | 0.745642026 |
|  |  | Inverse variance weighted | 18.54694 | 7 | 0.0097315 |  |  |  |
| PD | PCSK9 | MR Egger | 8.994234 | 11 | 0.6224245 | -0.002422105 | 0.015386104 | 0.877765039 |
|  |  | Inverse variance weighted | 9.019016 | 12 | 0.7013054 |  |  |  |
|  | HMGCR | MR Egger | 0.997231 | 6 | 0.9857170 | 0.043096053 | 0.066509399 | 0.540991814 |
|  |  | Inverse variance weighted | 1.417095 | 7 | 0.9850419 |  |  |  |
| AD | PCSK9 | MR Egger | 11.01095 | 11 | 0.4423465 | 0.019209304 | 0.013594415 | 0.185313015 |
|  |  | Inverse variance weighted | 13.00959 | 12 | 0.3683443 |  |  |  |
|  | HMGCR | MR Egger | 3.957185 | 6 | 0.6824705 | 0.010699967 | 0.063475598 | 0.871675255 |
|  |  | Inverse variance weighted | 3.985601 | 7 | 0.7814344 |  |  |  |

PCSK9, proprotein convertase subtilisin/kexin 9; HMGCR, 3-hydroxy-3-methylglutaryl coenzyme A reductase; CHD, coronary heart disease;ALS,amyotrophic lateral sclerosis; PD, Parkinson's disease; AD, Alzheimer disease.
